# TL1A Induces TCR Independent IL-6 and TNF-α Production and Growth of PLZF^+^ Leukocytes

**DOI:** 10.1371/journal.pone.0085793

**Published:** 2014-01-08

**Authors:** Kirsten Reichwald, Tina Z. Jørgensen, Peter Tougaard, Søren Skov

**Affiliations:** Laboratory of Immunology, Faculty of Medical and Health Sciences, University of Copenhagen, Frederiksberg C, Denmark; INSERM-Université Paris-Sud, France

## Abstract

An elevated level of the cytokine TL1A is known to be associated with several autoimmune diseases, e.g. rheumatoid arthritis and inflammatory bowel disease. However, the mode of action of TL1A remains elusive. In this study, we investigated the role of TL1A in a pro-inflammatory setting, using human leukocytes purified from healthy donors. We show that TL1A, together with IL-12, IL-15 and IL-18, directly induces the production of IL-6 and TNF-α from leukocytes. Interestingly, TL1A-induced IL-6 was not produced by CD14^+^ monocytes. We further show that the produced IL-6 is fully functional, as measured by its ability to signal through the IL-6 receptor, and that the induction of IL-6 is independent of TCR stimulation. Furthermore, the transcription factor PLZF was induced in stimulated cells. These results offer a substantial explanation for the role of TL1A, since TNF-α and IL-6 are directly responsible for much of the inflammatory state in many autoimmune diseases. Our study suggests that TL1A is a possible target for the treatment of autoimmune diseases.

## Introduction

TL1A (TNF like Ligand 1A, encoded by TNFSF15) is a cytokine initially described as a T cell co-stimulator, signaling through the receptor DR3 [1]. TL1A is increased in several autoimmune diseases, such as rheumatoid arthritis (RA) [2]–[4], psoriasis [5], inflammatory bowel disease (IBD) [6], [7] and ankylosing spondylitis [1], [8], [9]. Several different studies have implicated a role of TL1A in the induction of Th17 related cytokines, although opinions differ on the exact role of TL1A [10]–[12]. Knockout of DR3 or TL1A in different autoimmune mouse models ameliorates disease and delays disease onset of both collagen-induced arthritis [13] and experimental autoimmune encephalomyelitis (EAE) [14]. DR3 is essential for the development of allergic lung inflammation [15], but also involved in protection against certain viruses and bacteria, demonstrating its complex role [16], [17].

IL-6 is recognized as a mediator of inflammation, both because of its direct stimulatory effect on B cells, but also because of the pro-inflammatory potential of the IL-6/sIL-6R complex [18]. IL-6, together with TGF-β, directly induces IL-17 production from pathogenic Th17 cells [19], and blocking of IL-6 has efficacy in RA treatment, in particular when anti-TNF treatment is inadequate [20]. Hence, IL-6 is directly involved in inflammation, but it also renders T cells less responsive to T_reg_ suppression via STAT3 activation [21], [22].

**Figure 1:**
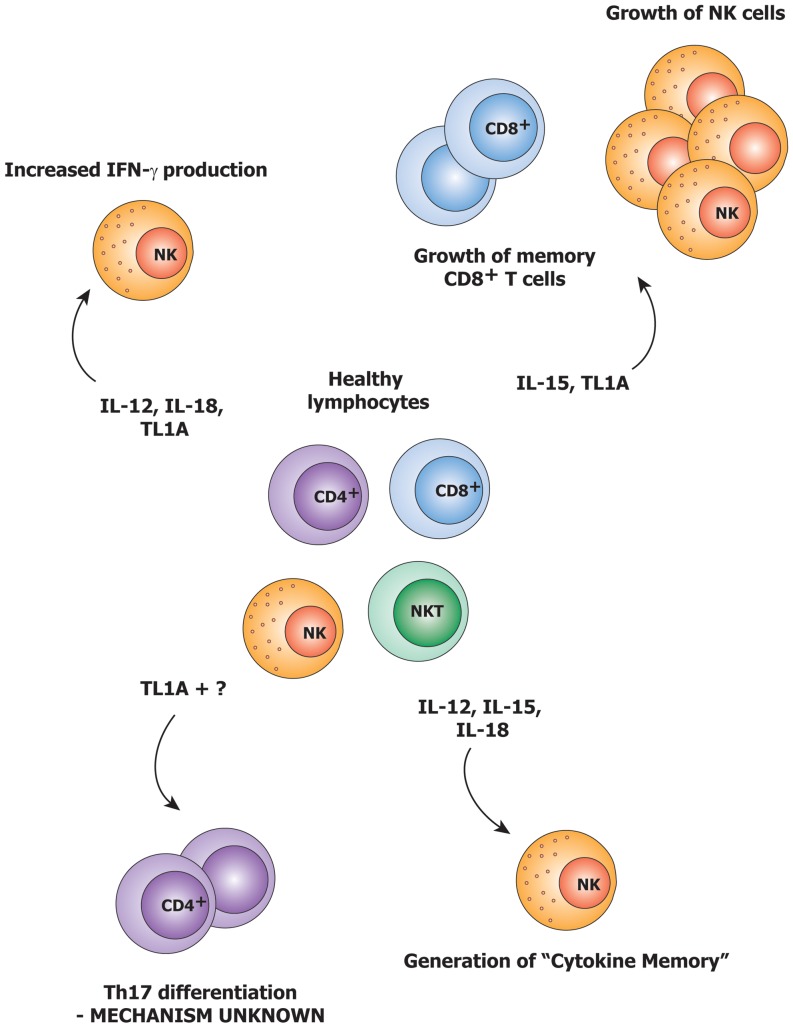
An overview of previous studies on the pro-inflammatory cytokines used in our setup. IL-15 is known to induce the growth of NK cells and memory CD8 T cells, and also causes CD8 T cells to acquire functional NK receptors [25], [56], [57]. IL-12 and IL-18 are known to induce IFN-γ in both NK and NKT cells in synergy with TL1A [23]. IL-12, IL-15 and IL-18 have recently been shown to induce “cytokine memory” [26]. TL1A is also known to be involved in Th17 development [10], [12], but the mechanism remains unidentified.

Different groups have shown that TL1A has the capability of synergizing with IL-12 and IL-18 in inducing IFN-γ in NK cells and other T cells [23], [24]. In addition, combinations of IL-12, IL-18 and IL-15 are known not only to induce IFN-γ [25], but also “cytokine memory” in NK cells. This cytokine memory is not clearly understood, but involves priming of NK cells by cytokines, resulting in enhanced IFN-γ production upon re-stimulation [26]. Furthermore, IL-15 has been shown to cooperate with TNF in the activation of dendritic cells [27], and to be involved in disturbing the T_reg_/Th17 balance [28], [29], which is a hallmark of autoimmune pathogenesis [10], [11], [21], [30]. IL-15 is known to augment memory T cell function [31], but also boosts their autoimmune capacity by inducing expression of classical co-receptors on activated CD4 T cells [32] and of NK receptors on CD8 T cells [33]. In **Figure 1**, we have summarized the effect of TL1A, IL-12, IL-15 and IL-18 on naïve lymphocytes.

Bystander activation and cytokine activation of different lymphocyte subsets is being intensely studied [34]–[37] due to its clear significance in orchestrating inflammatory responses. Initially, bystander activation of CD8 T cells or, to a lesser degree, CD4 T cells was described [38], [39], but by now cytokine activation is regarded an innate feature of many conventional T cells and innate-like lymphocytes. In fact, many lymphocytes might only need a combination of an appropriate STAT activator and an IL-1 family cytokine to initiate the production of cytokines [36].

PLZF (promyelocytic leukemia zinc-finger) is a transcription factor involved in stem cell maintenance [40]. It is critical for NKT cell and ILC development and directly induces effector functions in memory CD8 T cells. By now, it is recognized as an inducer of innate-like features in many different lymphocytes [41].

In this study, we show that TL1A in combination with IL-12, IL-15 and IL-18 directly induces antigen-independent IL-6 and TNF-α from monocyte-depleted PBMCs. Although we were unable to identify the exact cell type, we show that the IL-6 produced is functional and TL1A-dependent, and that the stimulation increases PLZF.

## Materials and Methods

### Purification of Lymphocytes

Buffy coats from healthy blood donors were obtained from the Blood Bank at the Copenhagen University Hospital (Denmark), in agreement with the local ethics committee (Region Hovedstaden). PBMCs were purified by Ficoll-Histopaque density gradient centrifugation. For PBLs, 200 µL washed pan-mouse beads (Invitrogen, Cat# 11041) were added to 2×10^8^ PBMCs in 30 mL complete RPMI, and left in the incubator for at least 1 h to allow phagocytic cells to take up beads or adhere to the plastic culture flask. PBLs were collected by removing cells attached to the beads with a magnet; remaining cells were used for subsequent stimulations. For TCR stimulation studies, PBMCs were plastic depleted twice to obtain PBLs. CD14 depletion was done according to manufacturer’s protocol using CD14 beads (Invitrogen, Cat# 11149D). For depletion studies, Dynabeads Pan Mouse IgG beads (Invitrogen, Cat# 11041) was used according to manufacturer’s protocol. Depletion was done using the same antibodies (same clone and manufacturer, but non-labeled) as used for flow cytometry, as listed below. For cytokine stimulation, cells were set up at a density of 10^6^/mL in complete RPMI and the following cytokine concentrations were used, unless stated otherwise in the figure: IL-12 (RnD Systems, Cat# 219-IL): 4 ng/mL, IL-15 (Peprotech, Cat# 200-15): 10 ng/mL, IL-18 (MBL, Cat# B003-5): 40 ng/mL, TL1A (RnD Systems, Cat# 1319-TL): 100 ng/mL, TL1A blocking Ab (RnD Systems, Cat# MAB7441): 1 µg/mL, tocilizumab (RoActemra, Roche): 10 µg/mL. For different TCR stimulations, plastic-depleted PBLs were stimulated with 4 µg/mL CD3 (eBioscience, Cat #14-0037), 25 µL/mL (according to the manufacturer) CD3/CD28 beads (Invitrogen, Cat # 111.32D) or 2.5 µg/mL SEA (Staphylococcal enterotoxin A (Sigma-Aldrich, Cat#S9399)). The supernatants were harvested on day 6, and bead-based ELISA was performed on the samples.

### Flow Cytometry

CFSE staining was done using 5 µM CFSE (Invitrogen, Cat# C34554) as described by Parish et al. [42] and stimulated with cytokines for 6 days prior to flow cytometric analysis. Antibodies used: CD8-APC (BD, Cat# 345775), CD3-APC (BD, Cat# 555335), CD4-APC (Biolegend, Cat# 300514), CD56-APC (eBioscience, Cat#17-0569-42), CD16-PE (Biolegend, Cat#302007), HLA-DR-PE (Biolegend, Cat# 307605), NKG2D-APC (RnD Systems, Cat# FAB139A). For intracellular staining, IL-6-PE (eBioscience, Cat#12-7069) and IFN-γ-FITC (BD, Cat# 554700) were used. Isotype controls were IgG1 APC (BD, cat # 555751), rat IgG1-PE (Invitrogen, Cat # R104) and IgG1-FITC (BD, Cat #555748). PBMCs were incubated for 6 h +/− LPS (Sigma-Aldrich, L2654, 1 µg/mL) and cells were stained using the BD Cytofix/Cytoperm Kit (BD, Cat# 554714), Golgistop (BD, Cat# 554724) or Golgiplug (BD, Cat# 555029) according to the manufacturer’s protocol. Samples were run on an Accuri C6 Flow cytometer and data were analyzed using FCS Express v. 3.

### Functional Assay

For the functional testing of IL-6, PBMCs from healthy donors were purified as described above, plated at 5×10^6^ in a thin layer, and left to adhere for 1 h. The supernatant was quickly removed by suction, and one of the following were added: complete RPMI containing either (1) nothing (control), (2) recombinant human IL-6 (1, 10 or 50 ng/mL, Peprotech, Cat# 200-06) or (3) the supernatant from CD14-depleted cells incubated with IL-12, IL-15, IL-18 and TL1A as described above, harvested after 6 days (IL-6 concentration: 27 ng/mL). As a control, tocilizumab (RoActemra, Roche, 8 µg/mL) was applied to two samples with either supernatant or rhIL-6 (10 ng/mL). After 30 min, these solutions were quickly removed by suction, and cold RIPA buffer containing 1 mM EDTA and phosphatase/protease inhibitors (ThermoScientific, Cat# 78441) was added. The cells were extracted for western blot as described below.

### Western Blot

For the both functional assay and for the whole cell PLZF blot, samples were extracted with ice cold RIPA buffer including phosphatase/protease and 1 mM EDTA. Samples were left on ice for 15 min, centrifuged for 10 min at 15,000×g and the supernatant collected. For the cytoplasmic/nuclear extract, cells were harvested on day 4 and extracted as described by the manufacturer using the Nuclear Extract Kit (Active Motif, Cat # 40010). Samples were run on 4–12% Bis-Tris gels (Novex, Cat# WG1401BX10) using MOPS buffer (Invitrogen, Cat # NP0001) with an antioxidant (Invitrogen, Cat# NP0005). Proteins were blotted onto a nitrocellulose membrane (Invitrogen, Cat# IB3010-01) using iBlot from Invitrogen, program P3. The membrane was blocked using a 2% Blotto solution (Santa Cruz Biotechnology, Cat# SC-2325,), washed in PBS/0.1% Tween (except for the pSTAT3 membrane, for which TBS/0.1% Tween was used) and incubated with shaking overnight at 4°C with the primary antibody. Primary antibodies used were: PLZF (1∶1000, Santa Cruz Biotechnology, Cat# sc-28319), STAT3: (1∶2000, Cell Signaling, Cat# 4904p) and pSTAT3 (1∶2000, Cell Signaling, Cat# 9145p). After washing again, the secondary Ab was added: swine anti-rabbit HRP (1∶1000, Dako, Cat# P0399) or rabbit anti-mouse HRP (1∶1000, Dako, Cat# P0260) and the membrane was incubated with shaking at RT for 1 h. After washing, the SuperSignal West Pico Chemiluminescent Substrate (Thermo Scientific, Cat# 34080) was added onto the membrane and left for 5 min before developing the films (CarestreamKodakBiomax light film, Sigma-Aldrich, Cat# Z373508-50EA). Blots of STAT3 were performed on the same membranes as those used for pSTAT3. Membranes were stripped for 25 min in Restore PLUS Western Blot Stripping Buffer (Thermo Scientific, Cat# 46430), washed and re-blocked before detection as described above.

### Measurement of Cytokine Production

Cells from different setups were harvested at different time points (see figures) and centrifuged for 10 min at 1000×g. Supernatants were collected and used for measurement of cytokines either by bead-based ELISA or standard ELISA kits. For several cytokines, bead-based ELISA kits were used: Diacone Diaplex Th1/Th2/inflammation, Nordic Biosite, 880 100 010. For IL-6 only: Diacone Diaplex, Nordic Biosite, 880 030 001. Both bead-based assays were run on the Accuci C6 flow cytometer, and data were analyzed using the Flowcytomix Pro software (eBioscience).

## Results

### TL1A Induces Proinflammatory Cytokines

TL1A synergizes with IL-12 and IL-18 in the activation of NK, NKT and T cells [23], [24], and IL-15 has a synergic effect on IL-12 or IL-18 [25]. Since TL1A is elevated in several chronic inflammatory diseases [2], [5], [7], [9], we wanted to investigate the range of cytokines regulated by TL1A. Freshly purified PBMCs were incubated with a combination of different cytokines, and the production of cytokines was measured after 6 days of stimulation. We used a multiplex approach, enabling us to measure 10 different cytokines in one sample (IFN-γ, IL-1β, IL-2, IL-4, IL-6, IL-8, IL-10, IL12p70, IL-17A and TNF-α).

**Figure 2:**
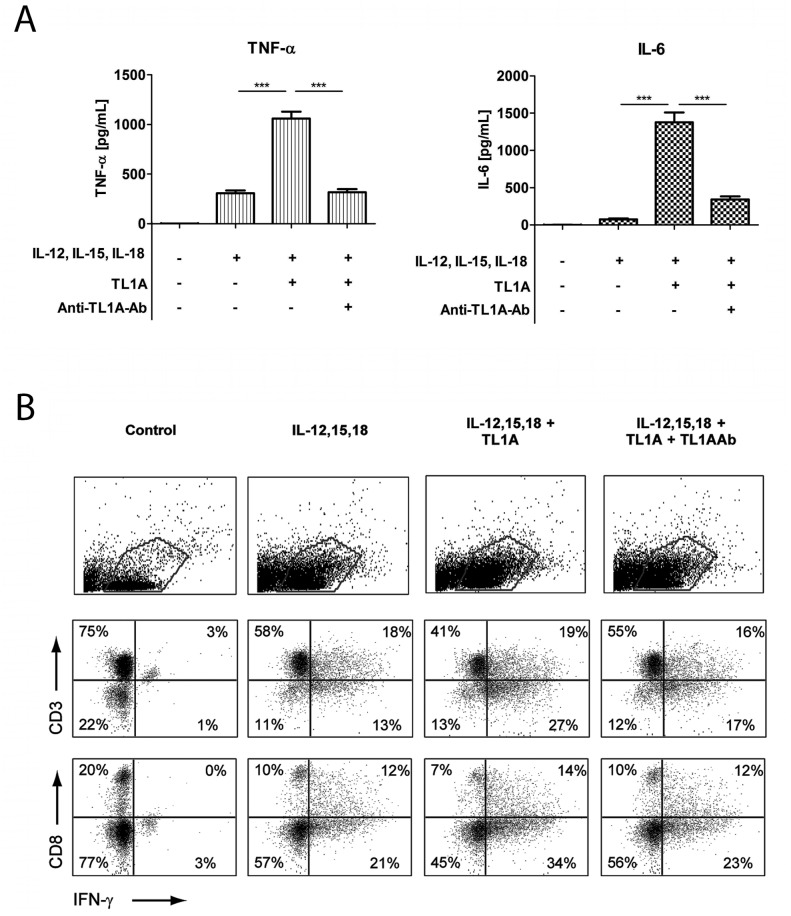
TL1A induces IL-6 and TNF-α. Freshly purified PBMCs were incubated with IL-12 (2 ng/mL), IL-15 (10 ng/mL), IL-18 (10 ng/mL), TL1A (100 ng/mL) and TL1AAb (1 µg/mL, blocking antibody). Extra IL-15 (2 ng/mL) was added on day 3. (**A**) After 6 days, supernatants were collected and different cytokines were measured by bead-based ELISA. Error bars represent the SEM of eight measurements. Statistically significant differences are indicated by ***(t-test, P<0.001). Data are representative of three different experiments with cells from three separate donors (**B**). After 6 days, cells were stained extracellularly for CD3 or CD8 and intracellularly for IFN-γ and analyzed by flow cytometry as described in the Materials and Methods. The upper panels show gating for lymphocytes; the two lower panels show CD3/IFN-γ and CD8/IFN-γ staining. Data are representative of three different experiments with cells from three separate donors.

As shown in Figure 2a, the cytokine measurements clearly showed a strong induction of IL-6 and TNF-α by TL1A, whereas classical stimulatory cytokines such as IL-2 and IL-4 were not detected (data not shown). Addition of a TL1A blocking Ab completely abolished the production of IL-6 and TNF-α, demonstrating both specificity and integrity. Also, the blocking effect of anti-TL1AAb ruled out the possibility that the TL1A effect was caused by endotoxin or other impurities.

We also measured IFN-γ in PBMCs after 6 days of cytokine stimulation (Figure 2b) using intracellular staining. As expected, the combination of the different cytokines along with TL1A resulted in a strong induction of IFN-γ, as has been shown previously [23], [25], [26].

### IL-6 is Rapidly Induced in Monocyte-depleted PBL Cultures

Since monocytes and macrophages are known as the main source of IL-6 in the initiation of inflammation [43], we decided to investigate if monocytes produced IL-6 after stimulation with IL-12,IL-15, IL-18 and TL1A.

**Figure 3:**
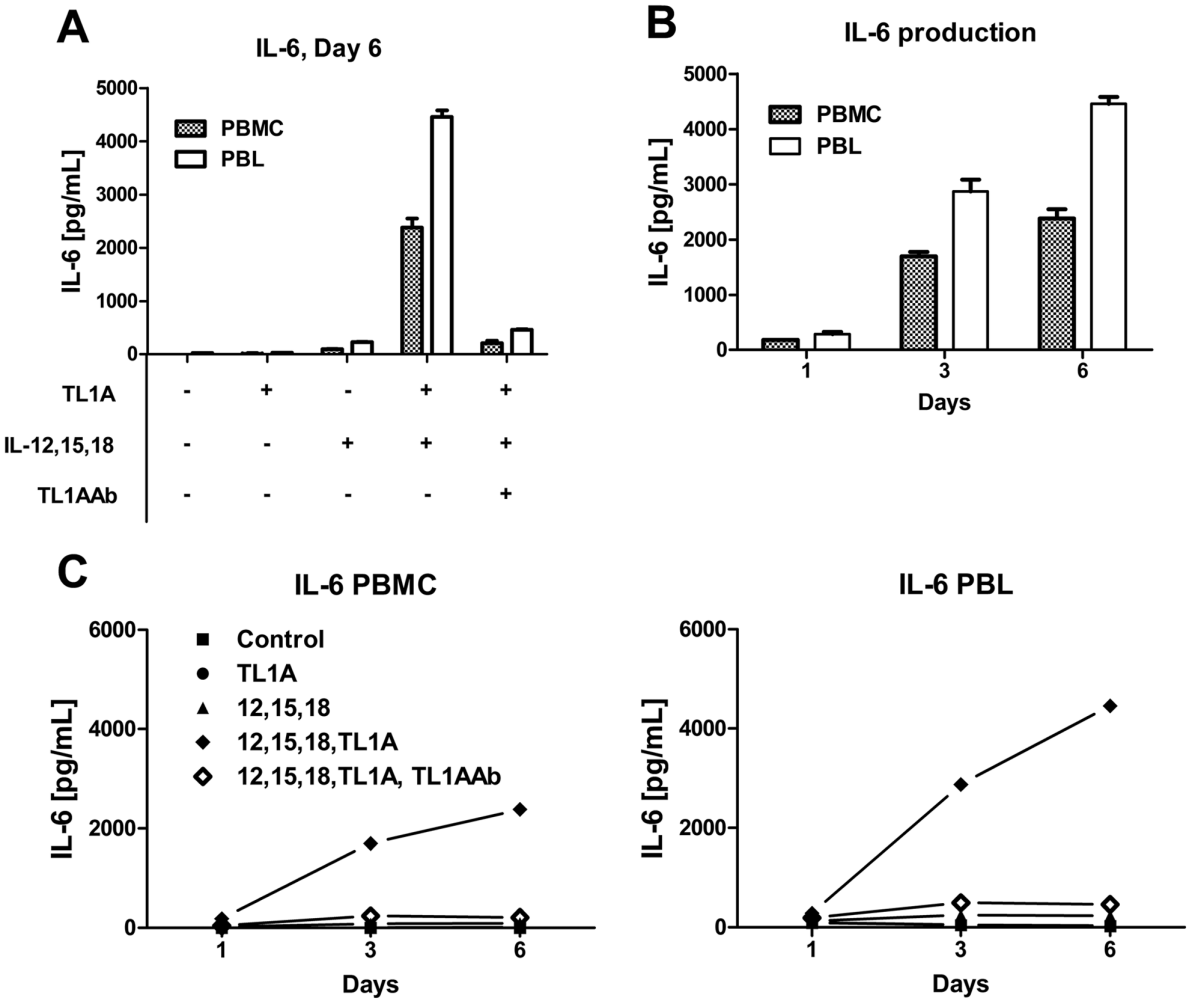
IL-6 production is higher in PBLs than in PBMCs. PBMCs and PBLs were prepared as described in the Materials and Methods, stimulated for 1, 3 or 6-12 (4 ng/mL), IL-15 (10 ng/mL), IL-18 (40 ng/mL), TL1A (100 ng/mL) and TL1AAb (1 µg/mL) and IL-6 was measured in the supernatants. (A) PBMCs and PBLs were stimulated with different combinations of cytokines and IL-6 was measured on day 6. (B) IL-6 production by PBMCs and PBLs stimulated with IL-12, IL-15, IL-18 and TL1A for 1, 3 and 6 days. (C) IL-6 production after 1, 3 and 6 days by PBLs and PBMCs stimulated with different cytokine combinations. Error bars represent the SEM of two measurements. Data are representative of three different experiments with cells from three separate donors.

**Figure 4:**
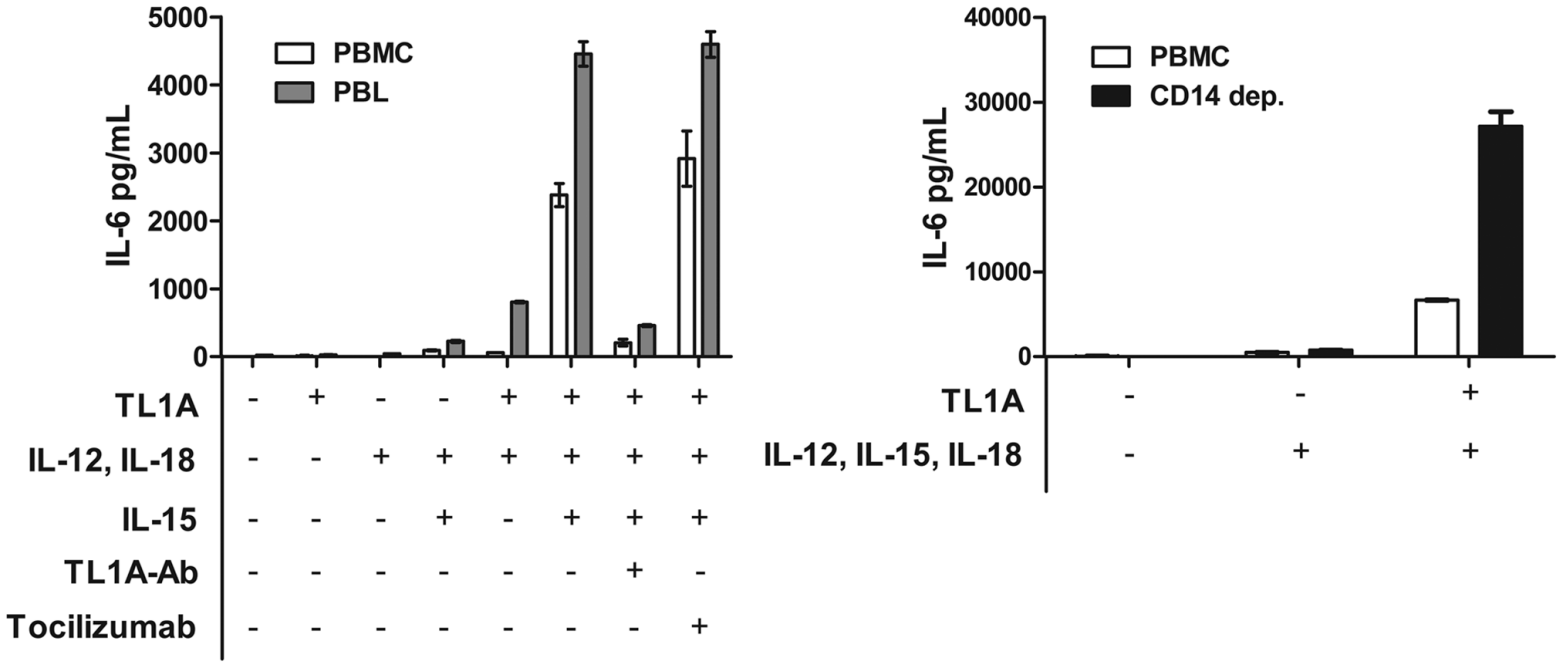
Depletion of CD14^+^ monocytes enhances TL1A-dependent IL-6. PBMCs, PBLs and CD14-depleted PBMCs were prepared as described in the Materials and Methods, stimulated for 6 days with combinations of IL-12 (4 ng/mL), IL-15 (10 ng/mL), IL-18 (40 ng/mL), TL1A (100 ng/mL), TL1AAb (1 µg/mL) and tocilizumab (RoActemra, 8 µg/mL), and IL-6 was measured in the supernatants. Error bars represent the SEM of two measurements. Data are representative of three different experiments with cells from three separate donors.

We incubated both PBMCs and PBLs with different combinations of cytokines and measured IL-6 production after 1, 3 and 6 days. As shown in **Figure 3**a, neither TL1A nor the combination of IL-12, IL-15 and IL-18 were able to induce any IL-6 on their own. PBLs stimulated with IL-12, IL-15, IL-18 and TL1A produced higher levels of IL-6 than stimulated PBMCs (4400 pg/mL vs. 2300 pg/mL). The combination of IL-12, IL-18 and TL1A also induced IL-6 production in PBLs, as shown in **Figure 4**, but the levels were several fold higher when IL-15 was also added (800 pg/mL vs. 4400 pg/mL). The data shown in **Figure 3**b and **Figure 3**c demonstrate that IL-6 production was already high at day 3, and higher in PBLs than in PBMCs.

Hence, when comparing stimulated PBLs with PBMCs, monocytes were not the source of IL-6 (**Figure 3**). To verify this, we stimulated CD14-depleted PBMCs (**Figure 4**). The results presented in **Figure 4** confirmed that IL-6 was in fact not produced by monocytes, as much higher levels were obtained when stimulating CD14-depleted cells. This shows that IL-6 was produced by CD14-depleted leukocytes only by stimulation with a pro-inflammatory cocktail of TL1A, IL-12, IL-15 and IL-18. To confirm that the difference in IL-6 production between PBLs and PBMCs was not due to uptake of IL-6 by monocytes, we added IL-6R blocking tocilizumab, which did not result in any difference in IL-6 production (**Figure 4**).

### TL1A-induced IL-6 is Functional

**Figure 5:**
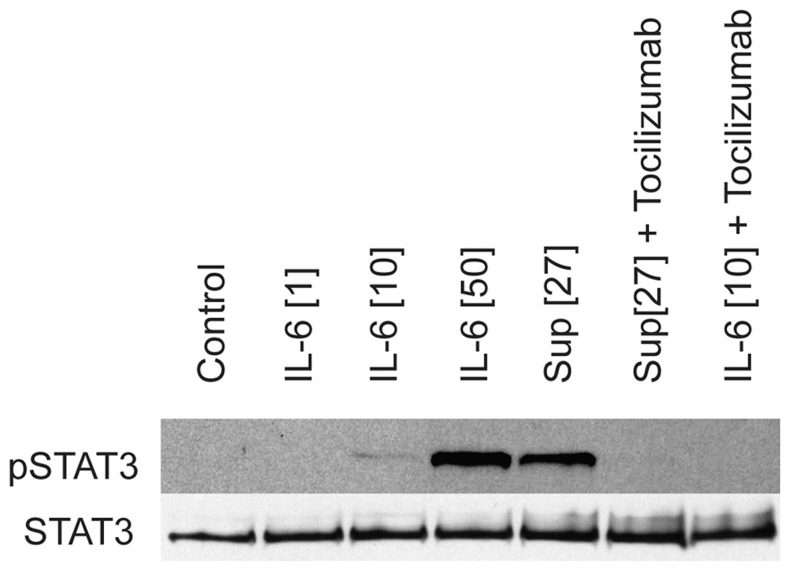
TL1A-induced IL-6 phosphorylates STAT3. A functional pSTAT3 was set up to verify the functionality of TL1A-induced IL-6. In short, purified PBMCs were incubated for 30-6 (1, 10 or 100 ng/mL) or the supernatant from cells incubated with IL-12 (4 ng/mL), IL-15 (10 ng/mL), IL-18 (40 ng/mL), TL1A (100 ng/mL); the supernatant contained 27 ng IL-6/mL (Sup [27]). PBMC proteins were extracted and pSTAT3 and STAT3 were detected by western blotting as described. Additionally, to verify that IL-6 was in fact the cytokine responsible for STAT3 phosphorylation, both the supernatant (27 ng/mL) and rhIL-6 (10 ng/mL) were added along with tocilizumab (IL-6R blocking Ab). Data are representative of three different experiments using cells from three separate donors and supernatants from two different trials.

It is known that IL-6 signaling results in STAT3 phosphorylation, which inhibits the suppression of T cells by T_regs_ [21]. To validate the function and integrity of the IL-6 produced, we tested the ability of the collected supernatant to phosphorylate STAT3, using rhIL-6 as a positive control. In short, we purified PBMCs from healthy donors and monocytes enriched by plate adherence. We added media with either rhIL-6 or IL-6 from a supernatant of the cytokine stimulated PBLs. After 30 min, the supernatant was removed and cells were harvested for western blot detection of STAT3 and pSTAT3, as shown in **Figure 5**. To confirm that phosphorylation was indeed mediated by IL-6, the IL-6R antagonist tocilizumab (RoActemra) was added in two separate samples to block signaling from both the supernatant and rhIL-6 (**Figure 5**). The results clearly demonstrate that the IL-6 produced was functional, and that IL-6 signaling was essential for STAT3 phosphorylation.

### The Effect of TL1A is TCR Independent

**Figure 6:**
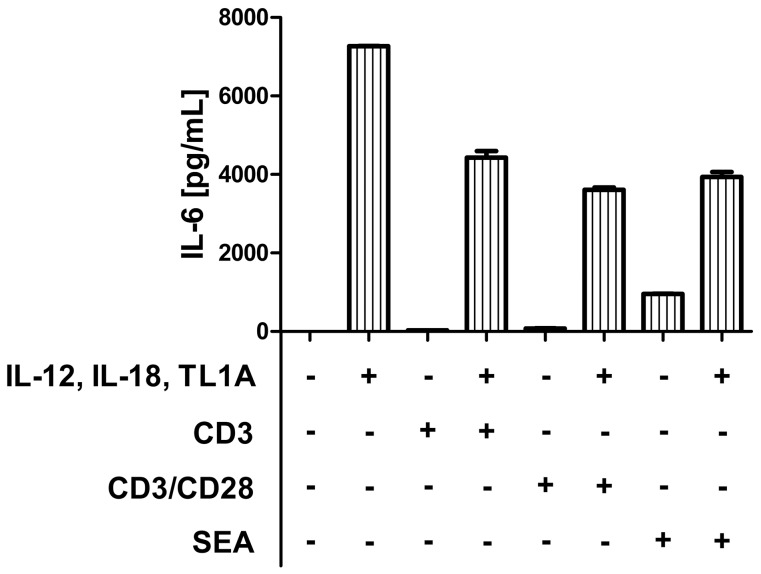
IL-6 production is TCR independent. PBLs were TCR stimulated using anti-CD3 (4 µg/ml), CD3-28 beads or SEA (Staphylococcal enterotoxin A, 2.5 µg/ml). The supernatants were harvested on day 6, and bead-based ELISA was performed on the samples. Error bars represent the SEM of two measurements. Data are representative of three different experiments using PBLs from three different donors.

To investigate if the IL-6 production was affected by TCR stimuli, we set up an experiment using different combinations of IL-12, IL-18 and TL1A +/− TCR stimuli. In short, PBLs were stimulated with cytokines (IL-12, IL-18 and TL1A), TCR stimuli (Staphylococcal enterotoxin A (SEA), CD3 or CD3/CD28) or both. After 6 days of stimulation, the supernatant was collected and IL-6 was measured. As shown in **Figure 6**, the combination of IL-12, IL-18 and TL1A resulted in the production of IL-6, as expected. TCR stimulation in general did not result in IL-6 production, although SEA did induce low levels of IL-6 by itself.

The induction of IL-6 was still observed after TCR co-stimulation, although there was a tendency that TCR stimulation downregulated IL-6 production. This indicates that the imprinting achieved by the cytokines is not substantially affected by TCR signaling.

### Intracellular IL-6 Staining

The most obvious question now was: exactly which cells are IL-6 producers? IL-6 is routinely measured intracellularly in monocytes after stimulation, and we therefore tried to measure IL-6 by intracellular flow cytometry. We could not detect TL1A-induced IL-6 in cultured PBLs or PBMCs by intracellular staining. We tried different time points (ranging from 6 to 72 h after addition of cytokines) and different inhibitors of intracellular transport and lysosomal function. First, we tried both Golgistop (contains monensin, which blocks transport from the Golgi/ER) and Golgiplug (contains brefeldin A, which blocks exit from the ER) +/− PMA/ionomycin, without success. We speculated that this could be due to the cells using different secretory lysomes than those used in the classical ER/Golgi pathway, and so tried two different lysosomal inhibitors: bafilomycin A (inhibits fusion between autophagosomes and lysosomes) and chloroquine (prevents fusion of endosomes and lysosomes). Still, we did not succeed in measuring intracellular IL-6 in leukocytes (data not shown).

**Figure 7:**
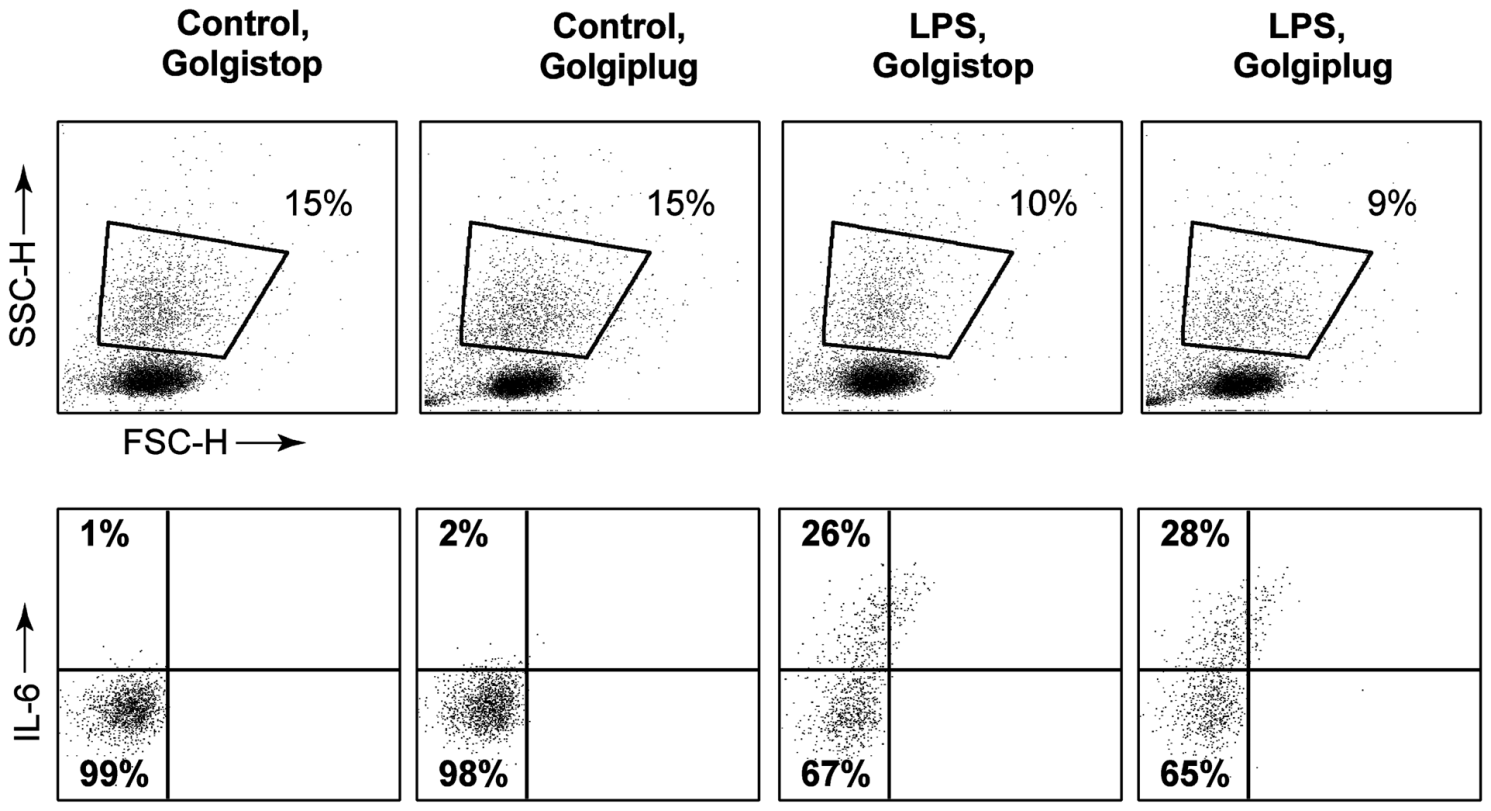
Intracellular IL-6 staining is possible in monocytes. Freshly purified PBMCs were incubated for 6+/− LPS (100 ng/mL) and Golgistop or Golgiplug. Cells were stained intracellularly for IL-6, and analyzed by flow cytometry. Upper panels: monocyte gating for control and LPS samples with Golgiplug/stop. Lower panels: % of gated cells positive for IL-6 expression. Data are representative of three different experiments using PBMCs from three separate donors.

To verify our intracellular IL-6 procedure, we set up PBMCs incubated with LPS and Golgistop/plug for 6 h (**Figure 7**). This clearly showed that the method applied was not per se flawed, as we could readily measure IL-6 produced by stimulated monocytes. The difficulties in measuring TL1A-induced intracellular IL-6 and possible explanations for this are further reflected on in the Discussion.

### Characterization of Leukocytes Induced by TL1A

**Figure 8:**
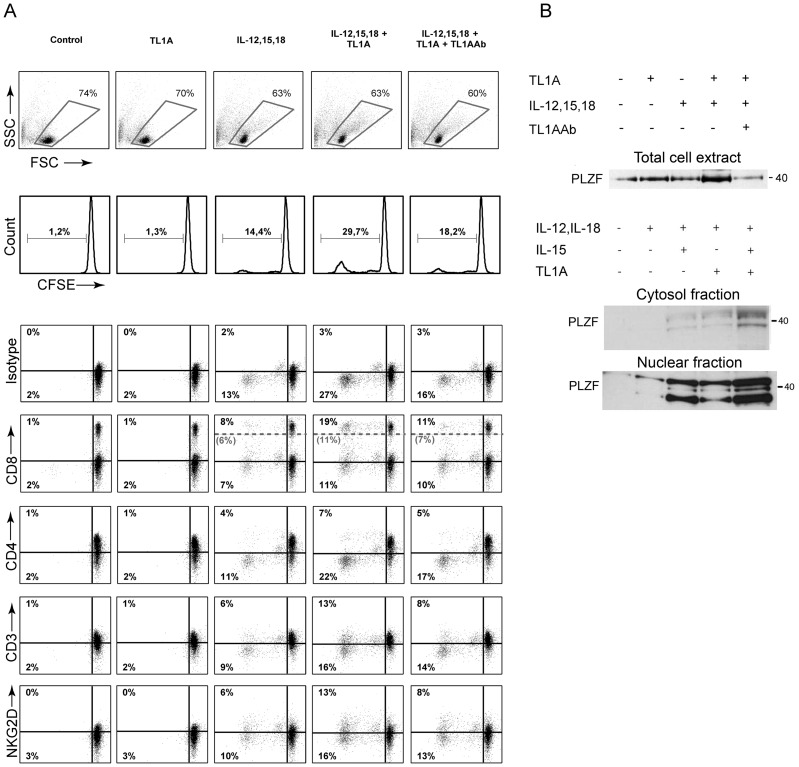
TL1A induces the growth of PLZF^+^ cells. (A) PBLs were purified from healthy donors, CFSE stained and stimulated for 6 days with combinations of IL-12 (4 ng/mL), IL-15 (10 ng/mL), IL-18 (40 ng/mL), TL1A (100 ng/mL) and TL1AAb (1 µg/mL). Upper panel shows gating on live cells and (%) proliferating cells as seen by less CFSE/cell. Lower panels show staining of different surface molecules. Note that for CD8, both CD8^+^ (bright+dim) and CD8^dim^ cells are shown (intermediate (%) in grey); see text for elaboration. Data are representative of three different experiments with cells from three separate donors. (B) PBLs were extracted after 6 days of stimulation with the cytokines listed, and PLZF was detected in whole cell extracts or cytoplasmic/nuclear extracts. Data are essentially representative of three different experiments.

To see if TL1A and the cytokine cocktail had an effect on the proliferation of different leukocytes, we stained PBLs with CFSE [42], added cytokines as listed in **Figure 8** and observed the phenotype of growing cells after 6 days of stimulation.

It was evident from the proliferation data (**Figure 8**a) that TL1A had a strong synergistic effect when added with IL-12, IL-15 and IL-18. Alone, the combination of IL-12, IL-15 and IL-18 resulted in 14% proliferating cells after 6 days, whereas the addition of TL1A increased this to 30%. Interestingly, TL1A had no effect on its own, indicating that DR3 costimulation is essential, but not alone sufficient for proliferation.

The cells proliferating were to a large degree either CD8^bright^ (8%) or CD8^dim^ (11%), and partially CD3^+^ (13%) and NKG2D^+^ (13%). Hence, the cells were most likely a mix of CD8 T cells, NKT and NK cells. Almost no CD4 T cells proliferated. Again, the TL1A Ab effectively blocked the proliferative effect of TL1A (**Figure 8**a).

Along with this, we decided to detect the transcription factor PLZF (promyelocytic leukemia zinc-finger) using western blot. PLZF was initially described as being involved in stem cell maintenance [40], and is critical for NKT cell and ILC development [41], [44]. PLZF is also able to induce effector functions in memory CD8 T cells, and drives IL-17 production in CD8 cells, along with the stimulation of subtypes of γδ T cells and other innate-like lymphocytes without antigen stimulation [45]–[48]. As shown in **Figure 8**b, PLZF was clearly induced in cells treated with the combined cytokine stimulation, indicating NKT or other innate-like leukocytes.

**Figure 9:**
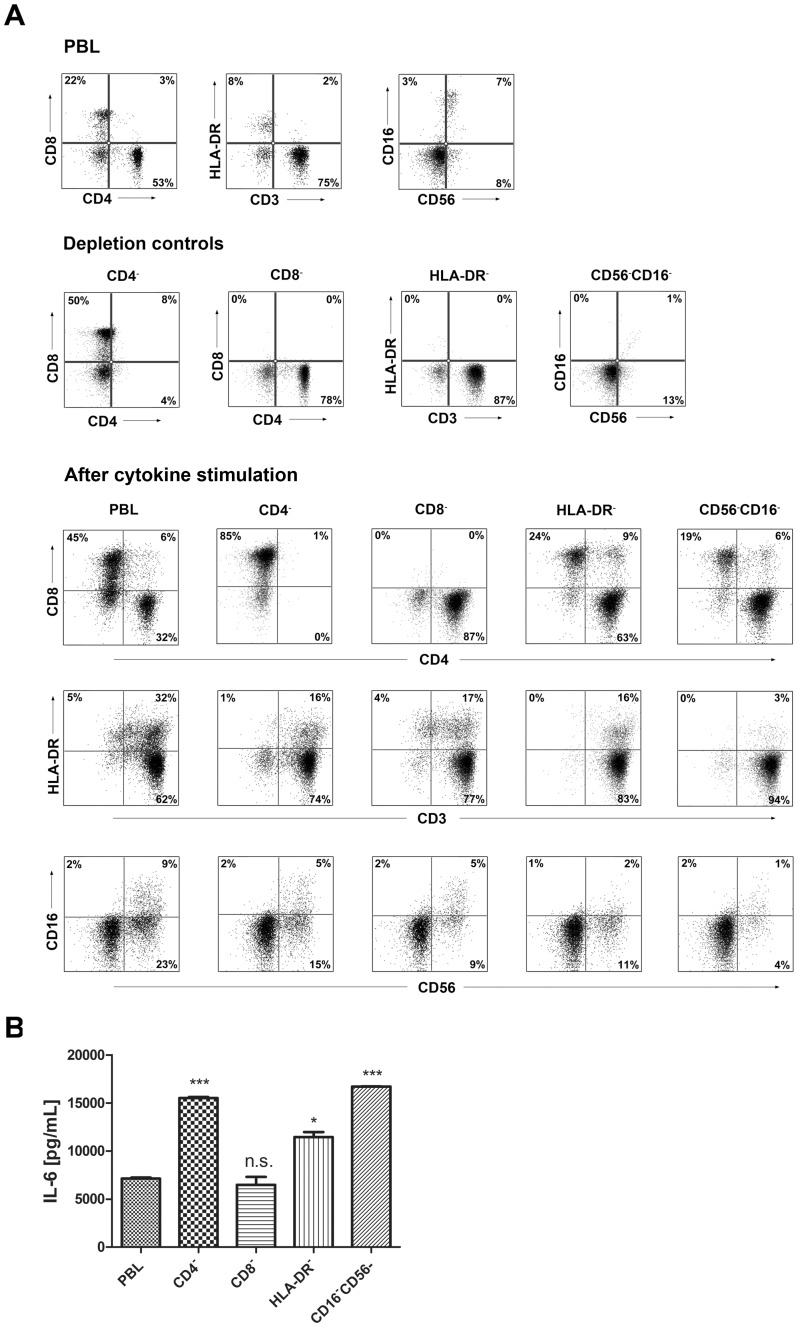
IL-6 produced by PBLs depleted for CD4^+^, CD8^+^, HLA-DR^+^ or CD56^+^/CD16^+^ cells. (A) Freshly purified PBLs stained for CD4, CD8, CD3, HLA-DR, CD16, CD56. Depletion controls for PBLs depleted for CD4^+^, CD8^+^, HLA-DR^+^ or CD16^+^/CD56^+^ cells. PBLs and depletions were stimulated for 7 days with combinations of IL-12 (4 ng/mL), IL-15 (10 ng/mL), IL-18 (40 ng/mL) and TL1A (100 ng/mL) and stained for CD4, CD8, CD3, HLA-DR, CD16 and CD56 expression. (B) IL-6 production after 7 days by PBLs and depletions stimulated with IL-12, IL-15, IL-18 and TL1A as described above. Error bars represent the SEM of two measurements. Statistically significant differences by t-test: *** = p<0.001, * = p<0.05, n.s. = not significant. Data are representative of two different experiments with cells from two separate donors.

To identify the IL-6 producing cell, we also tested different depletions. We targeted the cell surface markers CD4, CD8, CD16/CD56 and HLA-DR, assuming that these would cover classical CD4/CD8 T-cells, mature NK and NK T-cells and B-cells/dendritic cells. We then set up stimulations of the depletions and measured IL-6 production on day 7 and analyzed cells by flow cytometry. From the flow cytometric analysis shown in **Figure 9**a it became clear that even though the depletions were successful, cytokine stimulation led to differentiation of CD3^+^HLA-DR^+^ in the HLA-DR-depleted cells (from 0% to 16%). Furthermore, none of the depletions had targeted the IL-6 producing cell, as observed in **Figure 9**b, since all depletions displayed equal or higher IL-6 levels after 7 days of stimulation. Both CD4-, HLA-DR- and CD56/CD16-depleted cells produced significantly higher levels of IL-6 than non-depleted PBLs.

## Discussion

We have shown that TL1A, together with a pro-inflammatory cocktail of IL-12, IL-15 and IL-18, specifically induces TNF-α and IL-6 from CD14-depleted leukocytes. It is particularly interesting that IL-6 induction was much higher in CD14-depleted cells, since this proinflammatory cytokine is traditionally derived from monocytes. TL1A is elevated in the serum and synovial fluid of RA patients [2], [4], in psoriatic skin lesions [5] and in Crohn’s disease [6], [7]. Both IL-6 and TNF-α have well-described effects on both vascular function and stimulation of proinflammatory leukocytes. Hence, the link between TL1A and induced inflammation is apparent.

TL1A alone or the cytokine combination of IL-12, IL-15 and IL-18 did not result in IL-6 production, demonstrating the need for two different signaling events, similar to that observed for cytokine activation of ILCs [36]. Importantly, TL1A with IL-12 and IL-18 alone was sufficient for the induction of IL-6 production. However, IL-15 increased the level by several fold, and was therefore used in most setups. TL1A is known to synergize with IL-12 and IL-18 in IFN-γ production by NK and NKT cells [23], and IL-15 is known to augment IFN-γ production by NK cells when stimulated with IL-12 [26]. Hence, the effect of IL-15 could be to support indirect cytokine activation of a range of innate-like lymphocytes.

We tried several approaches for detecting the IL-6 producing cells, and were initially quite surprised that this was such a difficult task, since IL-6 is easily detected in LPS-activated monocytes. However, no matter the approach, intracellular IL-6 remained undetectable in our setup. There are several possible explanations for this:

1. It is well-known that staining for intracellular cytokines will only reveal proteins residing in the ER/Golgi. However, TL1A-induced IL-6 might be transported through an alternative pathway, e.g. lysosomes, that are harder for antibodies to access, regardless of permeabilization. However, we tried an assay for this pathway using lysosomal inhibitors before intracellular staining.
2. IL-6 might not be fully folded intracellularly, especially if the protein is present inside lysosomal compartments.
3. IL-6 producing cells are fragile and are lost during handling.
4. IL-6 might be expressed as a different splice variant, only containing some of the epitopes recognized by our antibodies. IL-6 does have smaller splice variants, but most of these have reduced biological activity.

Even though the IL-6 producing cells remained elusive, the produced IL-6 was functional, specifically in its ability to signal through the IL-6R. STAT3 phosphorylation and IL-6 signaling are known to interfere with the delicate balance between T_reg_ and T_eff_ cells [21], [22], a phenomenon often observed in autoimmune disease [22], [49], [50], supporting the pro-inflammatory potential of TL1A.

We mainly observed growth of CD8^+^/NKG2D^+^/CD3^+/−^ cells, indicating NK or NKT cells. It was also clear that there was an induction of the transcription factor PLZF. Note that we tried different loading controls such as ERK and GAPDH (data not shown), but since we compared highly activated and proliferating cells with naïve cells, both of these were also increased. PLZF is known as being critical to the development of NKT cells [44]. In a transgenic PLZF mouse, however, T cells in general acquire a more memory-like phenotype, along with a bias towards IL-17 production [48], and PLZF positive cells induce the growth of CD8^+^ memory cells [47]. Hence, the presence of PLZF^+^ cells might simply be NKT cells, but in conjunction with the cytokine profile observed, this might also represent the activation of a broad range of innate-like lymphocytes.

The results from our depletion studies demonstrated that differentiation has to be taken into account when evaluating depletion studies. Although we successfully depleted HLA-DR-positive cells prior to cytokine addition, the stimulation caused significant up-regulation of HLA-DR^+^CD3^+^ cells due to activation. We were able to show that the IL-6 did not derive from classical CD4^+^ or CD8^+^ cells, and not from mature NK/NKT cells or circulating HLA-DR positive cells. Hence, the source of IL-6 might be unknown precursor, making it difficult to remove them prior to stimulation.

Our results clearly demonstrate that TL1A in a pro-inflammatory environment is a potent mediator of cytokine activation. CD8^+^ T cells, NK cells and NKT cells have all been described as being affected by some of the cytokines applied with regards to growth and IFN-γ production. However, this is the first time that TL1A has been shown to directly induce IL-6 in cells from healthy donor cells.

There is no doubt that IL-6 plays an important role in several autoimmune diseases. Along with the soluble IL-6 receptor (sIL-6R), it has been shown to be involved in different forms of arthritis, inflammatory bowel disease, psoriasis, asthma and colon cancer [22], [51]–[53]. This knowledge has paved the road for antibodies targeting IL-6R or gp130 (to which the IL-6R binds for signaling) that are now introduced as part of the treatment strategy in many of the above mentioned diseases.

**Figure 10:**
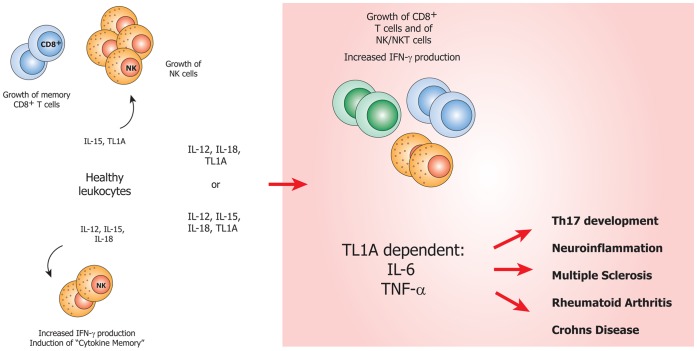
Proposed role of TL1A in the induction of inflammation and Th17 pathogenesis. IL-15 is known to support the growth of CD8^+^ T cells and NK cells [56], [58]. The combination of IL-12, IL-15 and IL-18 induces memory-like NK-cells [26]. We show that TL1A, in combination with IL-12 and IL-18 or IL-12, IL-15 and IL-18, specifically induces TCR-independent TNF-α and IL-6. Both are pro-inflammatory cytokines known to be key players in the development and progression of several autoimmune diseases.

Anti-IL-6R treatment is often successfully applied when anti-TNF has no effect, which is the case for 20–40% of RA patients [20]. This demonstrates that targeting TNF, although being a major leap in RA treatment, might not be the most effective strategy. TL1A was shown in this study to mediate the activation of different cells, resulting in the production of key inflammatory cytokines. One might therefore think of TL1A as an interesting target for RA treatment. Although this may be true, TL1A/DR3 signaling is critical to the viral immune response [16] and to optimally protect against Salmonella [17]. Hence, blocking TL1A might result in similar adverse effects as seen with anti-TNF and anti-IL-6R therapies [54]. Nonetheless, TL1A still represents an interesting target which seems to be upstream of both IL-6 and TNF, as illustrated in Figure 10.

We believe that future treatment strategies for autoimmune diseases such as RA and psoriasis will not only target cytokines, but the balance between regulatory/inflammatory cells in general. For example, helminths are currently being tested in clinical trials treating multiple sclerosis, due to their ability to alter disordered immune regulation [55]. This rather unspecific approach clearly shows us that we do not yet have a sufficient understanding of the regulation and imprinting of the immune system in autoimmune diseases. Nevertheless, the first step towards such an understanding is to move up the chain of events from the classical pro-inflammatory cytokines. Since TL1A directly induces TNF-α and IL-6, blocking it could be part of the solution in new treatment regimens.
